# Prediction of infarction volume and infarction growth rate in acute ischemic stroke

**DOI:** 10.1038/s41598-017-08044-4

**Published:** 2017-08-08

**Authors:** Saadat Kamran, Naveed Akhtar, Ayman Alboudi, Kainat Kamran, Arsalan Ahmad, Jihad Inshasi, Abdul Salam, Ashfaq Shuaib, Uvais Qidwai

**Affiliations:** 1 The Neuroscience Institute (Stroke Center of Excellence), Hamad General Hospital, Medical Corporation, Doha, Qatar; 2 Weill Cornell School of Medicine, Doha, Qatar; 3 Rashid Hospital, Dubai, UAE; 4 School of Liberal Arts, University of Illinois, Chicago, USA; 5 Shifa International Hospital, Islamabad, Pakistan; 6 Stroke Program, Department of Neurology, University of Alberta, Edmonton, Alberta, Canada; 7 KINDI Center for Computing Research, Qatar University, Doha, Qatar

## Abstract

The prediction of infarction volume after stroke onset depends on the shape of the growth dynamics of the infarction. To understand growth patterns that predict lesion volume changes, we studied currently available models described in literature and compared the models with Adaptive Neuro-Fuzzy Inference System [ANFIS], a method previously unused in the prediction of infarction growth and infarction volume (IV). We included 67 patients with malignant middle cerebral artery [MMCA] stroke who underwent decompressive hemicraniectomy. All patients had at least three cranial CT scans prior to the surgery. The rate of growth and volume of infarction measured on the third CT was predicted with ANFIS without statistically significant difference compared to the ground truth [P = 0.489]. This was not possible with linear, logarithmic or exponential methods. ANFIS was able to predict infarction volume [IV3] over a wide range of volume [163.7–600 cm^3^] and time [22–110 hours]. The cross correlation [CRR] indicated similarity between the ANFIS-predicted IV3 and original data of 82% for ANFIS, followed by logarithmic 70%, exponential 63% and linear 48% respectively. Our study shows that ANFIS is superior to previously defined methods in the prediction of infarction growth rate (IGR) with reasonable accuracy, over wide time and volume range.

## Introduction

The temporal evolution of ischemic stroke lesion is a highly dynamic process. Research in animal stroke models suggest that pattern of infarction growth is stroke model-specific and the logarithmic growth pattern has been used to most commonly described it^1–6^. The calculation of the rate of tissue loss or prediction of infarction volume at a particular point in time after stroke onset depends on the shape of the growth function of the typical ischemic stroke and the pattern of infarction growth in human stroke is most frequently assumed to be linear^7^, or logarithmic^8, 9^. Ischemic stroke lesions evolve dynamically during the acute phase showing wide variations in the growth rate with no correlation between time and diffusion lesion volume^10^. There is significant inter-species difference in time to reach maximal infarct volume compared to humans^2, 5, 11^, likely related to the species-specific differences in the vascularity and size and structure of the cerebrum. The optimal time point to perform imaging to predict the size of the infarction is not known. There is risk with very early imaging as it may underestimate the stroke volume. These factors highlight some of the difficulties in predicting stroke volume.

Prediction of infarction volume [IV] and infarction growth rate [IGR] in acute ischemic stroke can have important therapeutic implications. Decompressive surgery in malignant middle cerebral artery (MCA) stroke trials has traditionally sought to limit the treatment to less than 48 hours based on infarction size on CT scan and clinical deterioration^12, 13^. The accurate selection of patients for decompressive surgery is a laborious process and the time from onset of symptoms to surgery is very frequently based on clinical deterioration and the size of the infarction. Approaches that utilize tissue-based markers, for example, IGR and IV may be more helpful in the determination of the timing of surgery. Quantification of infarction evolution can also be used to determine the efficacy of therapy and may also be used as a surrogate outcome measure for stroke trials^14^.

The purpose of this study was to investigate infarction growth pattern that predict lesion volume at variable time intervals in patients with large vessel occlusion in the anterior circulation. We used various infarction growth models described in literature^2–9^ and compared them with Adaptive Neuro-Fuzzy Inference System [ANFIS] a method that has not been previously used to predict infarct volume and growth rate.

ANFIS is a biologically inspired algorithm utilizing two very powerful features of brain that are fundamental to the learning process; pattern modeling and perceptive inference. The brain is a highly sophisticated pattern matching system that utilizes repeated exposures over a pattern being learnt. This develops pattern-related pathways that are later used as pattern memory. The perceptive inference is related to the decision making capability of brain in spite of approximate and not too accurate data, implying that the brain does not work with exact thresholds but a range of values around that threshold. These two features of brain are computationally described as two separate techniques; Artificial Neural Networks (ANN) and Fuzzy Inference System (FIS). Fuzzy logic is a solution to complex problems with diverse applications. The applicability of fuzzy logic is not limited to research only, but has been used in clinical neurology^15–17^, imaging^18^ and neurosurgery^19, 20^.

## Results

**Figure 1:**
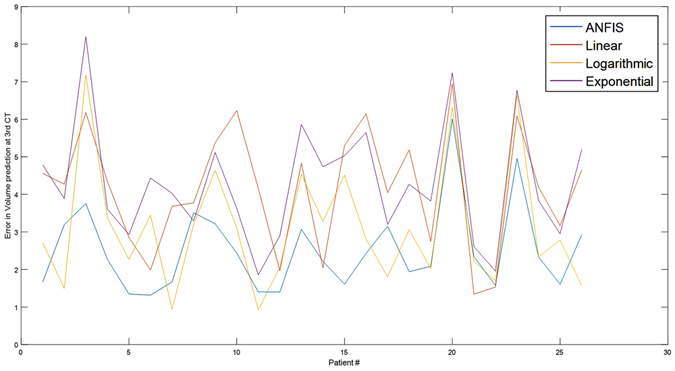
Comparison of mean squared of prediction error showing less errors by ANFIS compared to other prediction methods.

The pooled data had 137 patients who had undergone decompressive hemicraniectomy between 2007–2014. Sixty-seven patients had undergone at least three cranial CT scans prior to surgery. There was no statistically significant difference between training and testing data as regards patient age, risk factors, IV1 [P = 0.291], time of 1^st^ CT [P = 0.569], time of 2^nd^ CT [P = 0.615], time of 3^rd^ CT [P = 0.947], IGR1 [P = 0.428], IGR2 [P = 0.888] but IV2 was larger in training group [P = 0.020] [Tables 1 and 2 and Fig. 1].

**Table 1:** Data used for training ANFIS and testing for all methods used.

	Training data n = 41	Test data n = 26	P value
Age	50.95 ± 13.11	56.19 ± 12.023	0.105
Gender	34[82.9%]	24[92.3%]	0.465
Diabetes	12[29.3%]	9[34.6%]	0.646
Hypertension	22[53.7%]	19[73.1%]	0.112
Dyslipidemia	12[29.35]	12[46.2%]	0.160
Coronary artery disease	7[17.1%]	5[19.2%]	0.822
Congestive Heart Failure	4[9.8%]	2[7.7%]	0.773
Infarct Volume 1^st^ CT cm^3^	73.26 ± 76.30	75.08 ± 58.67	0.291
Infarct Volume 2^nd^ CT cm^3^	250.94 ± 114.55	218.62 ± 79.36	0.020
Infarct Volume 3^rd^ CT cm^3^	352.65 ± 108.18	—	
Time 1^st^ CT hours	6.39 ± 6.76	5.15 ± 5.77	0.569
Time 2^nd^ CT hours	37.26 ± 24.48	28.27 ± 29.97	0.615
Time 3^rd^ CT hours	74.11 ± 54.37	64.24 ± 68.01	0.947
1^st^ IGR ml/hr	5.61 ± 3.07	6.74 ± 3.61	0.428
2^nd^ IGR ml/hr	8.33 ± 6.75	9.46 ± 7.94	0.888
3^rd^ IGR ml/hr	4.94 ± 7.15	—	

Values are mean with percentage, mean age with standard deviation.

**Table 2:** Individual patient data of third infarct volume, original and predicted by various methods at time of CT3, CORR Cross correlation, and high order variability of predicted values by various methods used for IV3 prediction.

Age	Risk Factor	Vessel Occlusion	Original volume	ANFIS	Linear Model	Logarithmic Model	Exponential Model	Time to CT3
66	DM, CAD	MCA	326.32	307.32	126.19	272.7	82.15	21.3
38	HTN	MCA	348.82	407.58	205.59	334.54	236.17	48.3
53	HTN, DyL	ICA, MCA, ACA	650	600.84	506.22	445.11	360.03	64.3
74	HTN	ICA, MCA, ACA	306.2	267.07	107.35	209.95	192.48	73.5
48	HTN, DyL, CAD	ICA, MCA, ACA	194.7	213.98	49.57	124.43	23.34	26.3
54	HTN	ICA, MCA, ACA	374.72	364.5	351.02	297.75	234.02	65.5
65	DM, HTN, DyL, CAD	ICA, MCA, ACA	272.4	249.16	123.23	265.25	59.31	56.3
52	HTN, DM, DyL, CAD, CHF	MCA	288.92	172.52	146.65	197.32	190.42	373.15
69	HTN, DM, DyL	MCA	390.48	327.67	163.19	242.27	196.09	49
54	HTN, DyL	MCA	336	372.48	531.1	266.35	235.62	110
82	HTN	ICA, MCA, ACA	135	163.7	305.96	120.35	56.9	50.5
52	DM, HTN, DyL	ICA, MCA, ACA	281.11	295.89	309.57	246.23	206.05	81.4
50	DM, DyL	MCA	406.38	352.34	252.12	274.78	123.86	37
60	DM, HTN	MCA	321.21	352.98	348.33	387.2	73.91	22
54	HTN	ICA, MCA, ACA	374.6	359.26	134.17	227.37	173.83	48.3
41	DyL	ICA, MCA, ACA	437.5	465.99	163.76	396.31	229.25	49.3
67	CHF	MCA	279.22	348.29	386.31	303.61	182.61	49
34	None	MCA	387.53	365.76	181.65	330.65	266.04	73
53	HTN, DyL	MCA	281.4	313.21	215.17	248.03	124.74	35.3
60	DM, HTN	MCA	479	268.16	663	236.89	35.04	32.4
35	None	ICA, MCA, ACA	217.44	266.79	198.55	262.16	132.69	42.36
51	DM, DyL	MCA	131.8	167.91	84.61	69.47	26.06	32.45
60	HTN, CAD	MCA	492.34	368.07	284.84	226.06	206.55	125
35	HTN	MCA	326.6	361.09	428.09	287.98	206.48	60.05
61	DM, HTN, DyL	MCA	220	244.11	68.78	121.23	102.56	53.4
60	HTN	ICA, MCA, ACA	372.6	318.78	211.04	357.78	146.5	29.35
		P Value	1.00	0.32	0.01	0.00	0.00	
		Skewness	0.57	0.69	0.96	−0.13	0.13	
		Kurtosis	4.03	4.49	3.32	2.92	2.60	
		CORR	1.00	0.82	0.48	0.70	0.63	

Infarct volume is in cm^3^, Time CT3 in hours. DM-diabetes mellitus, HTN-hypertension, DyL-dyslipidemia, CAD-coronary artery disease, CHF-congestive heart failure, ICA-internal carotid artery, MCA-middle cerebral artery, ACA-anterior cerebral artery.

Whereas ANFIS was able to predict the IGR3 and IV3 without any statistically significant difference compared to the ‘ground truth’ or original data, all other methods failed. No significant difference was found in the mean ANFIS-predicted and mean ground truth IGR3, 4.75 ± 3.67 ml/hr vs. 4.94 ± 7.15 ml/hr [P = 0.489] and mean ANFIS predicted and mean ground truth IV3 332.01 ± 112.21 cm^3^ vs. 352.65 ± 108.18 cm^3^ [P = 0.457] [Table 2]. Although natural logarithmic method performed better than the linear and exponential there was a significant difference in the predicted and original IGR3 and IV3 values [P = 0.00], followed by exponential [P = 0.00] and linear [P = 0.01] [Table 2] in comparison with the original data. The cross correlation [CRR] indicated similarity between the ANFIS-predicted IV3 and original data of 82%. This compared to70% for logarithmic 70%, 63% for exponential 63% and 48% for linear methods [Table 2].

Though ANFIS showed no statistically significant difference over all, there were six patients where a deviation from the ground truth IV was observed [Table 2, Fig. 1]. The difference was seen in two patients in whom a large change occurred between the second and third scans. In both patients the IV2 increased by >280 ml to reach IV3 in 19 and 75 hours [time CT3-time CT2] respectively, ANFIS underestimated the predicted IGR3 and IV3. In spite of a significant change in IV2 to IV3 of 170 and 200 in additional two patients, ANFIS error was not very significant. In one patient there with a time difference of 211 hours between CT2 and CT3 and another patient in whom the infarct grew only 24 ml over 29 hours, both patients had IGR2 < 0.8 ml/hr, and ANFIS over and under estimated the IGR and IV, respectively. Hence in patients with abrupt large change in infarct volume > 280 ml and very low infarct growth over 24 hours [<0.8 ml/hr.] ANFIS was not able to predict IGR3 and IV3 accurately. None of the methods used were able to predict IGR3 and IV3 on the six patients. However, in comparison to ANFIS, all other methods showed significantly larger errors [Fig. 1]. ANFIS was able to predict IV3 over a wide range of volume [163.7–600 cm^3^] and time [22–110 hours]. Although ANFIS was able to accommodate changes in IV of up to 200 cm^3^, it was unable to predict larger changes. Similarly at very slow growth [IGR of 0.8 ml/hr], ANFIS also failed to predict the IV3. In addition, the high order variability of ANFIS-predicted values was in agreement with the ground truth as shown by the skewness and Kurtosis [Table 2].

## Discussion

In our study we used IGR rather than absolute IV because growth rate is a more generalized parameter that maps infarction volume growth into a more compact range of values thus making it easier to model. This is because variability in IGR is smaller than that of IV and the information of volume and time can both be captured in IGR. Conventional mathematical techniques employ parametric data models such as linear, exponential or polynomial models. These models are likely not appropriate in evaluation of highly nonlinear stochastic data, essentially the type of data usually seen in growth and volume determination of ischemic stroke. The data extrapolated from primate and rodent brain is model specific^1, 2^. The high degree of variability of cerebral infarction in humans suggests that highly nonlinear data of human ischemic stroke may require more innovative methodology. We used ANFIS because it produces unconventional classification models and deals with the degree of truth and not true or false, also known as approximate reasoning. ANFIS hypothesize relationships within the data, and newer learning is able to produce complex characterizations of those relationships. Moreover, it is highly flexible and can accommodate a variety of complexities such as unequally spaced time points, non-normally distributed measures, complex nonlinear and time-varying covariates. Hence, ANFIS provides an alternative method to the difficult mathematical modeling of complex nonlinear problems and meets the mathematical modeling requirements of a system^21^.

Our data shows that ANFIS was the only method able to predict infarct growth rate and in turn infarction volume at variable time intervals without significant difference to the original data [measured IV3]. Except for the extreme changes in infarction volume or large time difference between imaging (leading to very slow IGR), ANFIS predicted IGR3 and IV3 across wider time and volume ranges. Our study also reveal that logarithmic model performed better than linear and exponential methods, however, none of the three standard methods were able to predict IV3 and IGR3 as close to the ground truth as ANFIS. Failure of ANFIS to predict IV and IGR in two patients and variations seen were due to the values being outside the bounds of data (statistical outliers). The error plots (Fig. 1) showed ANFIS to have the lowest number of errors compared to wider variations in error by the other three methods.

The dynamics of infarction growth in patients with large vessel occlusion has not been well documented although some studies report a natural logarithmic pattern^9, 22^. Human stroke may grow initially in a linear pattern followed by slower growth due to space limitation from cranium and dural attachments. In a primate stroke models, using longitudinal DWI, a natural logarithmic growth pattern has been reported in the acute stage of infarction^2^. The diffusion MRI based evolution of infarction in macaques has been shown to be closer to what has been observed in humans than in rodent models^4^. Our data supports these observations of infarction evolution following a natural logarithmic growth pattern but the cross correlation of natural logarithmic values with original data was only 70% compared to 82% with ANFIS. During the hyper-acute stage [1–6 hours] infarction growth can be mapped by both linear and natural logarithmic growth patterns^2^. The data from animal studies show that natural logarithmic growth can predict the infarction volume for up to 48 but not at 96 hours^2^. In the our study logarithmic model performed better in predicting IV in less than 48 hours, where as both exponential and linear models failed, similar to the studies in primate stroke models^2^. These results are however not shared in other reports using primate stroke models^4^. The infarction volume prediction beyond 48 hours yielded mixed results with all models except with ANFIS. ANFIS was able to predict infarction volume closest to the actual measured size.

All growth patterns [linear, logarithmic and exponential] except ANFIS fail to predict the lesion volume in the sub-acute stage [beyond 48 hours] of stroke. This is likely related to the time of maximal infarction volume and collateral circulation. In primate stroke models the maximal lesion volume is reached within 48 hours^2^. In rodents, the infarction growth likely stops within 3 to 6 hours of MCA occlusion^5, 23^. This compares to 70–74 hours in humans, indicating a significant species difference in growth patterns^24^.

It is a common clinical practice to base prediction models on an “average” patient with assuming similar clinical and anatomical characteristics. Unfortunately, this may be problematic when calculating the growth patterns for individual patients. The growth rate of early DWI lesions in acute stroke is highly variable as shown by the lack of correlation between time and diffusion lesion volume^10^. Lesions with poor collateral circulation will have rapid penumbral loss and will grow rapidly^11, 25^. In addition, the growth patterns may be affected by the presence of preexisting risk factors and concurrent medications, highlighting the difficulties in accurately predicting infarct growth.

**Figure 2:**
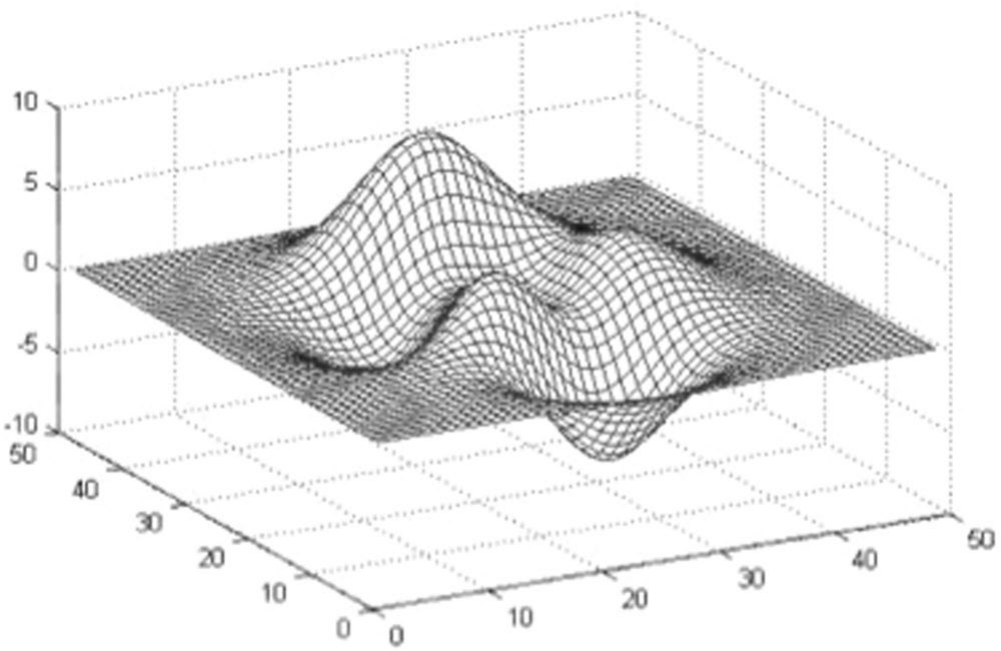
Typical constrained data set with dynamic values.

The use of non-contrast CT scan based data [IGR, IV] and ability to test the accuracy of various methods at the level of individual predictions over wide time ranges adds strength to our study. Nevertheless, prediction models have limitations. Our data shows that IGRs are quite variable with possible abrupt changes. These translate into dynamic changes that various infarction growth models are not able to capture. Infarctions in two patients in our series showed abrupt growths, over variable periods, where all growth pattern estimations failed to predict the IV3 and IGR3. The linear method, specifically, was unable to predict IV3, because the growth in linear model is based on a static slope that is calculated in the beginning only using the first two initial volume and corresponding time values. Hence, this slope value does not change throughout the stroke evolution and is not affected by the dynamic changes in the infarction evolution. A major shortcoming of the logarithm formula is that maximal infarction volume does not reach a plateau at the infinite point in time unlike the clinic scenario. Therefore, this pattern cannot be used for prediction of the infarct volume in the chronic stage^2^. The major disadvantage of ANFIS is that its accuracy limits are defined by the bounds of the data itself. We can understand ANFIS error by an example of a stretched sheet constrained on the ends so that it cannot be moved from the sides. However, the centre of the sheet can be stretched up or down to a limit to attain a maximum or minimum value [Fig. 2]. Hence, the positive and negative peaks cannot attain any value beyond a ‘stretchable’ limit of the function as defined by the underlying data set. In ANFIS, the error cancellation in the backward pass is similar to the peaks shown in Fig. 2. Hence, not all errors can be cancelled by counter gradient values. However, within specific limits of the data set, a large number of errors can be eliminated. In addition, the data training of ANFIS requires specialized software from ANN and fuzzy computation domains.

Our study has several limitations. First, our sample size may be small to reliably estimate the growth models. For ANFIS to eliminate this limitation, larger data sets are needed so that the correlated data implications can be modelled in stages rather than sudden large gradients (usually seen as outliers). Second the use of CT instead of DWI MRI, which is more sensitive for detecting ischemia, may have led to less accurate estimation of the first infarction volume. In routine clinical practice CT is the imaging modality used for repeat imaging rather than MRI due to cost and logistical issues as the patients are usually intubated and managed in intensive care units. Third, for the IGR measurement on CT scans, we presumed that CT changes were present when the stroke symptoms began. It is possible that the hypodensity developed at a later time interval; hence the first IGR may have a different value. We observed relatively large variations in standard deviation of the mean IGR. The wide variation in IGR has been observed by others as well and likely reflects the genetic variation in collaterals and the rate of collateral failure^11, 25, 26^. We speculate that the extreme changes in infarct volume that were not predicted by ANFIS may have been due to the failure of collateral circulation. The addition of collateral circulation status assessment may have improved ANFIS prediction. Fourth, during training we repeated Monte Carlo simulation 30 times based on our observation. We noticed that error did not change significantly after 25 iterations and a mature model stage had been reached.

In conclusion, we have shown that ANFIS can predict IGR and IV with reasonable accuracy, over wide time range while linear, natural logarithmic and exponential methods failed to predict IGR and IV. The prediction of IGR and IV, with large abrupt changes and extremes of growth remain undetermined even with ANFIS. Perhaps addition of collateral circulation to the predictive model will improve the results and extend its utility to less severe/smaller strokes and their growth pattern.

## Methods

We selected patients from our pooled decompressive hemicraniectomy [DHC] database from three tertiary referral centers in three countries [Hamad General Hospital, Qatar; Rashid Hospital, Dubai, UAE; and Shifa International Hospital, Pakistan]. Only patients with three brain-computed tomography [CT] scans during same hospital admission showing evidence of acute ischemia were selected. All patients had large vessel occlusion [ICA, MCA] on imaging. Patients were excluded if significant contralateral infarction or pre-existing infarction was present on the initial CT, only two imaging studies were performed or if imaging was uninterruptable, with parenchymal hematoma or hemorrhage with ventricular extension.

### Infarct Volume calculation [IV]

Measurement of the infarct volume [IV] was made using open source image analysis software OsiriX version 5.6^27^.

### Infarct Growth Rate calculation [IGR]

For first infarct growth rate [IGR 1] calculation we assumed the stroke volume to be zero prior to stroke onset.

Infarct growth rate 1[IGR1] = Δ volume (IV CT1–0)/Δ time (time CT1- stroke onset time)

Second infarct growth rate [IGR2] was measured on second CT [CT2]

IGR2 = Δ volume (IV CT2- IV CT1)/Δ time (time CT2-time CT1)

And third infarct growth rate [IGR3] was measured on third CT

IGR3 = Δ volume (IV CT3- IV CT2)/Δ time (time CT3-time CT2)

We used MATLAB 2015 for programming all prediction methods and for ANFIS, Fuzzy Logic toolbox was used in addition to core MATLAB coding environment.

For prediction of IGR3 and infarct volume on third CT [IV3], linear, natural logarithmic, exponential and ANFIS methods were used. The logarithmic and exponential equations have been used in previous publication^2^.

1. Linear method. For the linear method following equation was used

$${{\rm{V}}}_{3{\rm{L}}}={{\rm{V}}}_{2}+\frac{{{\rm{V}}}_{2}-{{\rm{V}}}_{1}}{{{\rm{t}}}_{2}-{{\rm{t}}}_{1}}({{\rm{t}}}_{3}-{{\rm{t}}}_{2})$$

2. Natural logarithmic method [ln]. For natural logarithmic function following equation was used

$${{\rm{V}}}_{3{\rm{G}}}={{\rm{V}}}_{2}+\frac{{{\rm{V}}}_{2}}{{{\rm{t}}}_{2}}\,\mathrm{ln}({{\rm{t}}}_{3}-{{\rm{t}}}_{2})$$

3. Exponential method [exp]. The exponential fitting was tested using the following equation

$${{\rm{V}}}_{3{\rm{E}}}={{\rm{V}}}_{2}+{{\rm{V}}}_{2}\,\exp \,[-(\frac{({{\rm{t}}}_{3}-{{\rm{t}}}_{1})}{100\,\mathrm{ln}(\frac{{{\rm{V}}}_{2}-{{\rm{V}}}_{1}}{{{\rm{t}}}_{2}-{{\rm{t}}}_{1}})})]$$

where *V*_1_: Infarct volume from 1^st^ CT scan, *V*_2_: Infarct volume from 2^nd^ CT scan, *V*_3*L*_: Predicted Infarct volume at *t*_3_ using Linear model (Equation 1), *V*_3*E*_: Predicted Infarct volume at *t*_*3*_ using Exponential model (Equation 2), *V*_*3G*_: Predicted Infarct volume at *t*_*3*_ using Logarithmic model (Equation 3), *t*_1_: Time at which 1^st^ CT scan was performed, *t*_2_: Time at which 2^nd^ CT scan was performed, and *t*_3_: Time at which 3^rd^ CT scan was performed.

4. Adaptive Neuro-Fuzzy Inference System [ANFIS].

**Figure 3:**
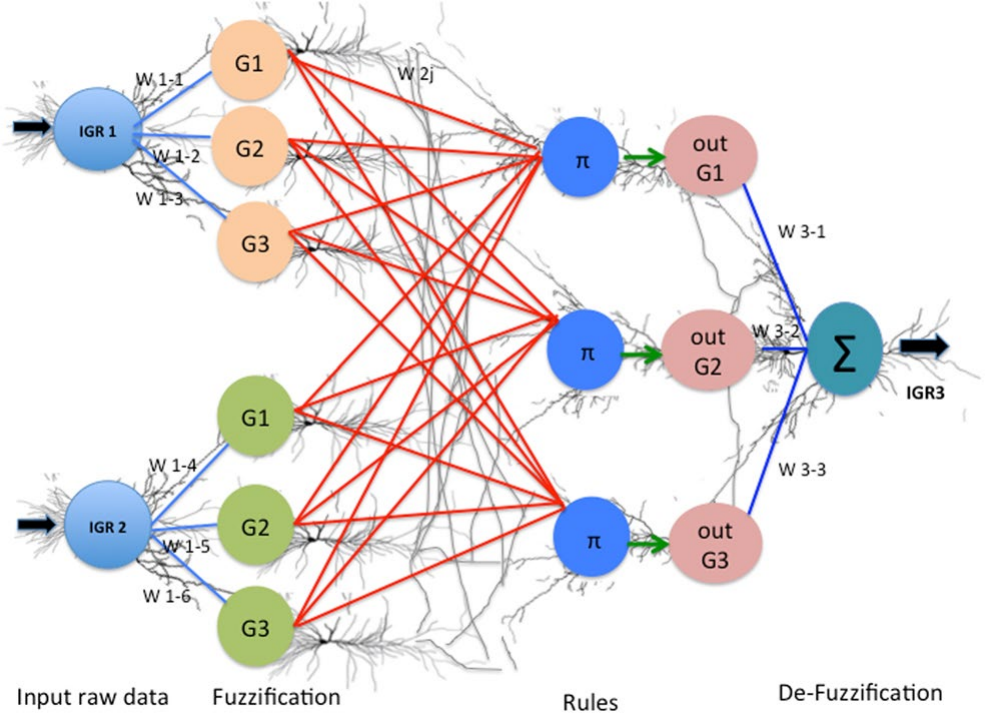
ANFIS structure and functioning explanation, superimposed on neurons to show the similarity between ANFIS and neuronal network structure and function.

ANFIS Artificial Neural Networks (ANN) and Fuzzy Inference System (FIS). ANFIS mathematically mimics the decision making process of the real neurons. In human brain, the learning process leads to the establishment of specific patterns of interconnections [dendrite-dendrite, dendrite-axon, axon-axon] made by a group of neurons. These interconnections are established in response to specific data inputs. The specific connections in the neuronal network excite (‘fire’) when a known pattern related to prior learning is received. In the same manner, ANFIS system mathematically groups the input data into clusters [like neurons] [groups G1, G2, G3 in Fig. 3] (through a process called Fuzzification).

The fuzzification of the input data is done by mapping the input data for the two inputs;IGR1 and IGR2, into three Gaussian membership functions [degree of truth and not absolute values] each named G1, G2 and G3 [also referred to as clustered data] Clustered data is not absolute numbers but the degree of conformity to the hypothesis i.e. IGR3 [Fig. 3]. The regrouping of these clusters, based upon their relevance to output groups [outG1, outG2, outG3, Fig. 3] is done through logical rules [Π in Fig. 3] Logical rules [Π in Fig. 3] are problem specific i.e. measurement of IGR3.

A set of following three logical rules [Π] connects the in the input [IGR1, IGR2] to the output, outG1, outG2, outG3 of IGR 3.

If (IGR1 is G1) and (IGR2 is G1) then (IGR3 is outG1)

If (IGR1 is G2) and (IGR2 is G2) then (IGR3 is outG2)

If (IGR1 is G3) and (IGR2 is G3) then (IGR3 is outG3)

**Figure 4:**
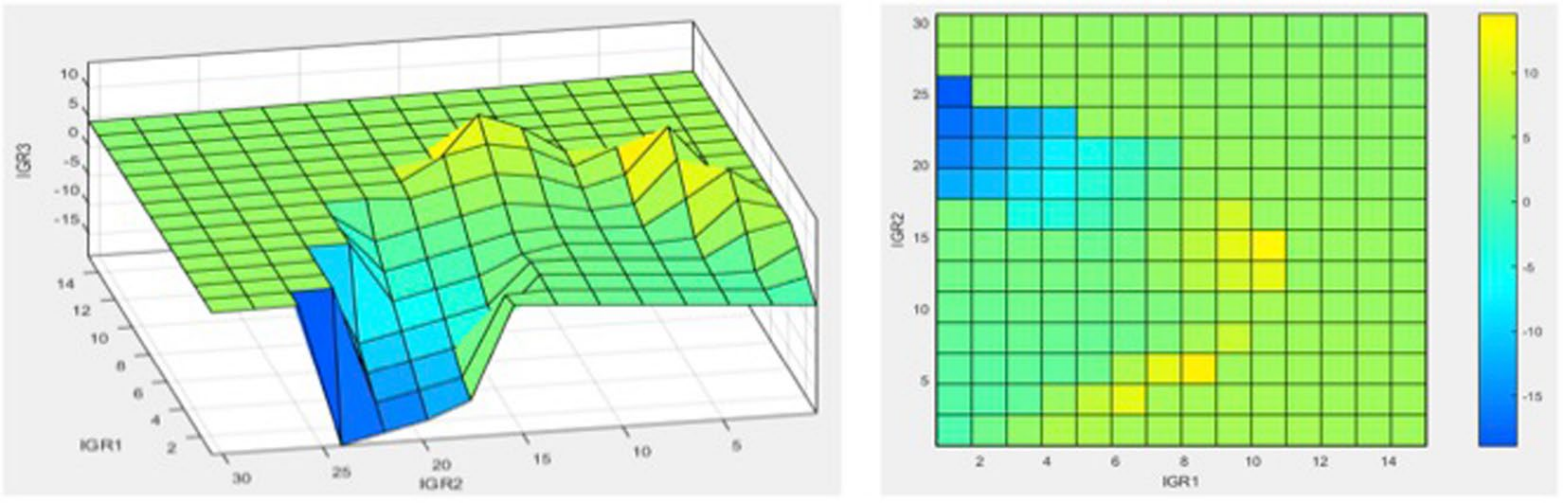
Final decision surface built by covering all the implications of the input data space. (**a**) 3D surface view, and (**b**) View from top [contour view]. X-axis represents IGR1 input; Y-axis IGR2 input while the output IGR3 is shown on the z-axis based on the above rules.

The logical rules [Π] represent the mathematical version of perceptive inference of brain also known as FIS [decision making capability of brain in spite of approximate and not too accurate data]. The information processing between input [IGR1, IGR2] and final output IGR3 is happening on clustered data [G1, G2, G3,][degree of truth or approximate data and not actual values] for both inputs [IGR1, IGR2]. However, the desired output [IGR3] has to be a discrete value. The output after rule application is still a cluster [comprising a range of values around the final IGR3]. To get the actual value of IGR3 from this cluster the centroid is calculated for the cluster. This process is called de-fuzzification [Σ in Fig. 3]. The centroid is a mean value of the cluster, which is the desired IGR3 actual value. Using the same procedure a decision surface was built by covering all the implications of the input data space [Fig. 4]. Each such centroid represents a grid-intersection point on the overall decision surface [Fig. 4].

The training process, tunes the connecting weights [shown in the figure as W], which are similar to the neuronal connections in brain. This is similar to the natural neurons making connections while being trained [dendrite to dendrite, dendrite to axon, axon to axon, axon to dendrite etc.].

ANFIS based parametric identification systems utilizes a hybrid learning rule combining the back-propagation gradient descent and a least-squares method. Initially, the input data is transferred through the network layers with initial weights and parameter values until they produce an output, called the Forward Pass. The generated output is then compared with the actual output from the data to calculate the error. Using the resulting error, the weights and control parameters are re-calculated, transferring the products of these gradients and error back to the input layer, called the backward pass. At any point, the weights are correlated through the output and error values simultaneously.

### Data training

The ANFIS system requires training on the type of data that will be later analyzed. Sixty percent of the data [41 patients randomly selected] was used for model training, using proposed prediction algorithm. To further enhance the performance, a Monte Carlo Simulation [MCS] method was repeated 30 times. In each repetition of Monte Carlo simulation 60% data was randomly selected, training and testing are repeated in the same fashion, mean squared error is calculated for each repetition and compared with previous one. The model with least mean squared error was kept for the next repetition so that at the end of MCS, the best model was obtained that covers all the modalities of the presented random data.

### Testing

After completion of training and developing best model, testing was performed on the remaining 40% [26 patients] data.

Following data was provided to all the methods used, IGR1, IGR2, time of CT1, time of CT2, and time of CT3 for the prediction IGR3 at time of CT3 [Table 1].

To calculate the IV3 from the output data [IGR3] following equation was used

$${{\rm{V}}}_{3}={{\rm{V}}}_{2}+{\rm{IGR}}3({{\rm{t}}}_{3}-{{\rm{t}}}_{2}).$$

where, V_3_ is the infarct volume3 at time t_3_ [time CT3], V_2_ is the infarct volume on second CT at time t_2_ [time CT2], and IGR_3_ is the predicted infarct growth rate at time t_3_ [time CT3].

### Statistical Methods

Statistical analyses were performed using Statistical Package for Social Sciences Version 22 (SPSS). Descriptive and inferential statistics were used to characterize the study sample and test hypotheses. Descriptive results for all quantitative variables (e.g. age) were presented as mean ± standard deviation (SD) (for normally distributed data). Numbers (percentage) were reported for all qualitative variables (e.g. gender). Bivariate analysis was performed using Independent sample t-test or Mann Whitney U-test whenever appropriate to compare all the quantitative variable (e.g. age, Infarct volume etc.) between training and test data groups. While all the qualitative variable (e.g. Gender, HTN etc.) between above two groups were compared by using Pearson Chi-square test or Fisher exact test as appropriate. A “P” value < 0.05 (two tailed) was considered statistically significant. The Cross correlation [CRR] was carried out between various methods of prediction. To calculate the high order variability of predicted values skewness and Kurtosis was also calculated.

### Compliance with Ethical Standards

The study adhered to the tenets of the declaration of Helsinki and was approved by the Institutional Review Board of Hamad Medical Corporation, Rashid Hospital, Dubai and Shifa International Hospital, Pakistan. Approval letters are enclosed with the manuscript.
